# The London Post-Graduate Course

**Published:** 1890-05-24

**Authors:** 


### Passing Topics.

### THE LONDON POST-GRADUATE COURSE.

The prospectus of the second series of lectures prepared by the committee of the "London Post-Graduate Course," is an eminently satisfactory document. The medical profession and the hospitals represented, together with humanity at large, may be alike congratulated upon the modicum of success already achieved in respect of a very important movement, at present in its infancy, but which is not unlikely to produce valuable and far-reaching consequences.

It is among the truisms that the practice of the special hospitals, in so far as it has been available for teaching purposes, has been allowed almost to run to waste, so that one of the two great functions assumed by hospitals has been but nominally performed in their case. True, the more eminent members of the staffs have utilised their opportunities by the publication of papers and books, or, when they have happened, as is usually the case, to be attached to one of the great general hospitals they may have disseminated the grains of knowledge garnered at the special hospital among the students of the school. It is obvious, however, that these are but imperfect methods, and, moreover, they are scarcely fair to the institutions concerned. We welcome the post-graduate teaching in the interest of all parties, and not least in that of the larger and reputable special hospitals, which now possess an additional credential to distinguish them from those useless and semi-private concerns with which they are sometimes confounded even in the minds of members of the profession.

But the chief value of this provision is found in the facilities afforded to the rank and file to keep themselves in touch with the leaders of their profession and to share the advantages of hospital work. Hitherto, a post-graduate in general practice has had no means of maintaining knowledge, or of making himself acquainted with the latest advances of science, except by reading books and medical journals, or by attendance at the meetings of the different medical societies. Now, he is enabled by the payment of a small fee and the expenditure of the necessary time to go through a regular and systematic course of demonstrations and lectures admirably designed to give him help, either in regard to the more difficult cases arising in his daily practice, or to enable him to acquire the higher degree, of which he may be laudably ambitious.

The names of the lecturers, and of the hospitals at which the lectures are to be given, are guarantees that the quality of the teaching will be of the best obtainable, and we note, as especially interesting features of the present course, that Bethlem Hospital is now added to the list of institutions represented, while, for the first time we believe, a poor law infirmary—Paddington—is made publicly available for tuition.

The institution of the London Post-Graduate Course has helped to rescue the greater special hospitals from the reproach so often cast upon them, though not always quite truthfully, by the representatives of the medical schools. It is not so long ago that even the foremost special hospitals scarcely dared to hold themselves forth as teachers, though a certain amount of good work of that kind was done *sub-rosâ*. Now, what with the attendance of practitioners in the wards and out-patients' rooms, and the lectures we are considering, we are inclined to think that the better special hospitals give promise of undertaking a very creditable share of medical education. These institutions so far from being hindrances to teaching, when used aright, really provide material and opportunities for the investigation of certain obstinate and obscure maladies, such as no general hospital can show. It was an American medical writer, who, a few months ago, stated that of all the cities in the world, London is pre-eminently the best for educational purposes, because of its splendid special hospitals. Let us hope that no ungenerous jealousy will limit to post-graduates the enjoyment of advantages meant for medicine's mankind.

Agricola.

